# Supplementary material for: Propagation of temporal and rate signals in cultured multilayer networks
Source: Nat Commun. 2019 Sep 3;10:3969. doi: 10.1038/s41467-019-11851-0 (PMC6722076; doi:10.1038/s41467-019-11851-0)
Supplement: Supplementary file 1 — Supplementary Information [file 41467_2019_11851_MOESM1_ESM.pdf]

Supplementary Information for

Propagation of temporal and rate signals in cultured multilayer networks

Jérémie Barral, Xiao-Jing Wang, and Alex Reyes

Supplementary Figures 1 to 19

Supplementary Table 1

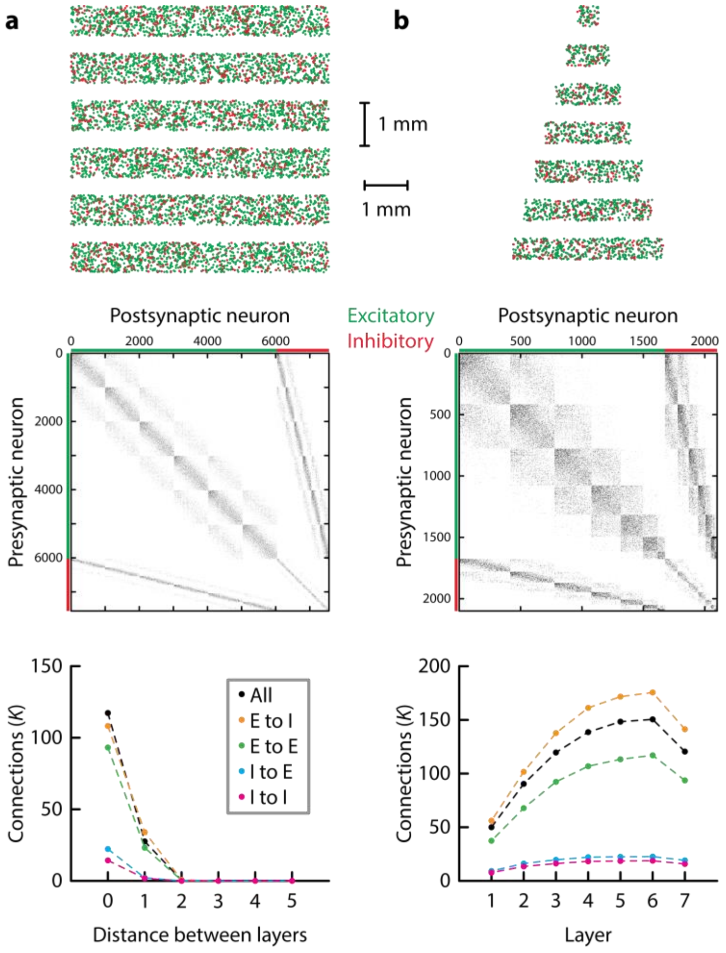

Supplementary Figure 1: Simulation of connectivity in multilayer networks.

We used simulations to compute the connectivity in multilayer networks made of layers of same size (**a**) or of different size (**b**). Top: schematic of the multilayer network. Excitatory (green, 80%) and inhibitory (red, 20%) neurons were placed randomly in delimitated chambers (**a**: length: 6 mm, width: 0.7 mm, spacing: 0.4 mm; **b**: length: [0.5; 1; 1.5; 2; 2.5; 3; 3.5] mm, width: 0.5 mm, spacing: 0.4 mm). The density was 300 neurons·mm^{-2}. Middle: we computed the connectivity matrix based on the following connection profiles: $P_c(x) = p_0 \cdot \exp(-x^2/2\sigma^2)$, where x is the distance between neurons and p_0 and σ were derived from experimental measures using dual patch-clamp recordings (*E*-*E*: p_0 = 0.4 and σ = 0.6 mm; *E*-*I*: p_0 = 0.6 and σ = 0.6 mm; *I*-*E*: p_0 = 0.6 and σ = 0.42 mm; *I*-*I*: p_0 = 0.5 and σ = 0.42 mm) [1]. Neurons were sorted by 1) their type (excitatory or inhibitory), 2) the layer they belong to, and 3) their position with respect to the center of the layer. Bottom: number of incoming connections K depended on the neuronal type and on the type of presynaptic neuron. The number of incoming connections was plotted as a function of the distance that separated neurons (**a**) or as a function of the position of the postsynaptic neuron in the network (**b**).

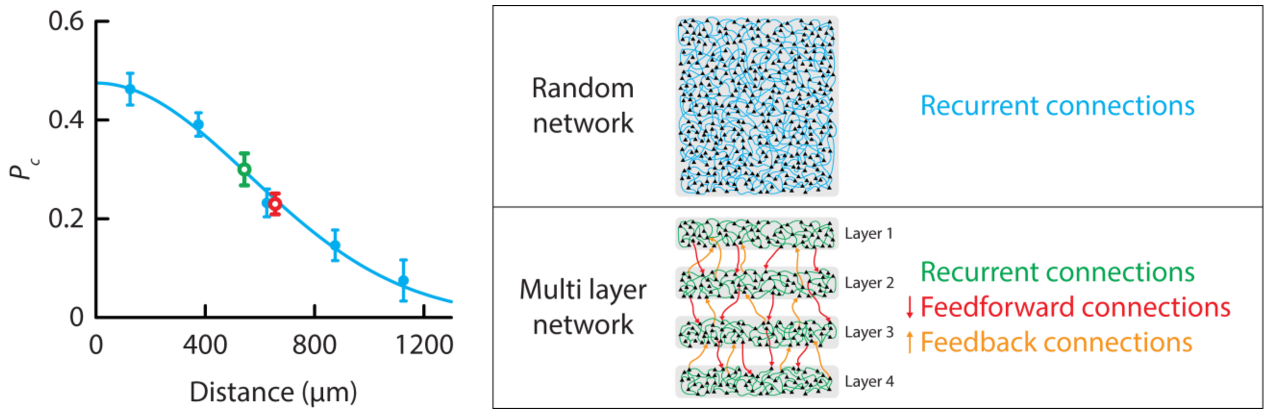

Supplementary Figure 2: Connectivity in multilayer networks.

Left, Plot of connection probability (P_c) vs intersomatic distance for an unstructured, random network (blue; data from [1]; schematic on right) and the Gaussian fit. Superimposed are the probabilities in multilayer networks for within layer recurrent connections (green; $P_c = 0.3 \pm 0.06$; mean ± SEM; n = 50 tested connections in 13 networks; 533 ± 132 µm apart; mean ± SD) and connections between two adjacent layers (red; $P_c = 0.23 \pm 0.04$, mean ± SEM; n = 100 tested connections in 13 networks; 666 ± 140 µm apart; mean ± SD). The data shows that connection probability did not depend on the architecture of the network and was readily predicted from the interneuronal distance. Also note that connection probabilities between and within layers were not statistically different (*t*-test statistics: $P = 0.170$). Data presented as mean ± SEM.

Right, schematic of connections in unstructured, random networks (top) and multilayer networks (bottom).

Note that the bidirectional connections between layers are often classified as feedforward and feedback though technically, whether a connection is feedforward or feedback depends on whether the stimulus is delivered in e.g. the first or last layer. Additionally, note that reciprocal connections were also observed with a probability that was slightly above chance level (0.12 ± 0.07 and 0.10 ± 0.04 for within and between layer connections, respectively).

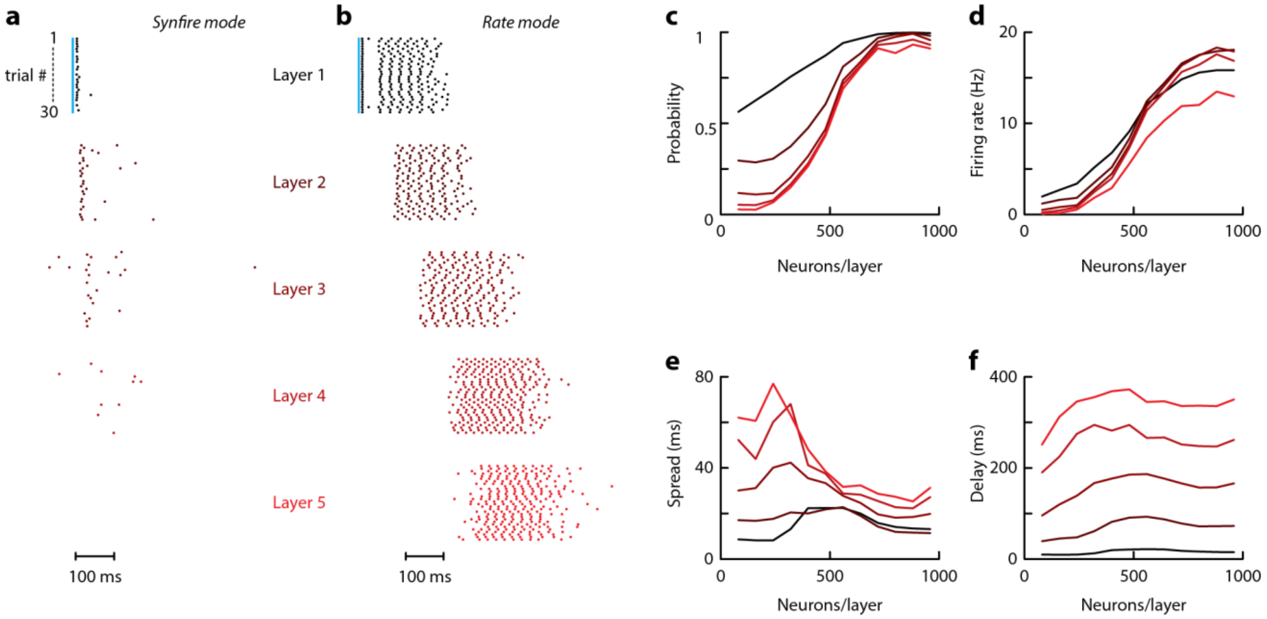

Supplementary Figure 3: Simulation of multilayer networks.

Multilayer networks were simulated as described in the Methods. Briefly, we used leaky integrate-and-fire neurons and conductance-based synaptic models. The networks consisted of 80% excitatory and 20 % inhibitory units. Excitatory synapses had a fast AMPA currents and a slower NMDA component. Intrinsic and connectivity parameters were derived from experimental measurements [1].

a-b, Examples of dot rasters showing spikes from individual neurons in each layer. Synchronized stimuli were delivered to one third of excitatory neurons in the first layer (30 repetitions of the stimuli). Data in **a** and **b** are, respectively, from sparse (100 neurons per layer) and dense networks (800 neurons per layer). **c**, Probability of evoking at least 1 spike vs network size for the different layers (layer 1 to layer 5 in black to red). **d**, Firing rate in the different layers vs network size. **e-f**, Temporal spread (**e**) and delay (**f**) of first spike vs network size for the different layers.

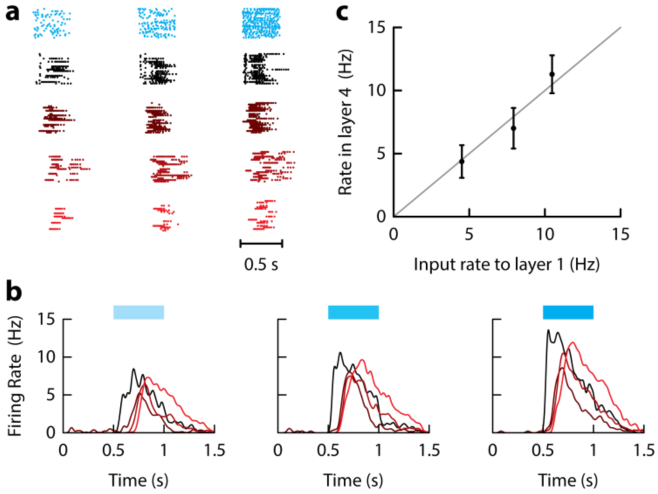

Supplementary Figure 4: Modulation of firing rate by the input firing rate.

a, Raster plot showing the 0.5 s-long Poisson trains of light pulses to the first layer with effective pulse rates of 5, 10, and 20 Hz (blue). Below are shown the responses of neurons in layer 1 to 4 (from black to red, respectively). **b**, Average PSTH compiled from 5 different networks for the 3 different input frequencies. **c**, Output firing rate in layer 4 vs firing rate of the delivered Poisson input the first layer. The original input rate (5, 10, and 20 Hz) was corrected for the decrease in efficacy of ChR2 at high rates [1]). Data are presented as mean ± SEM.

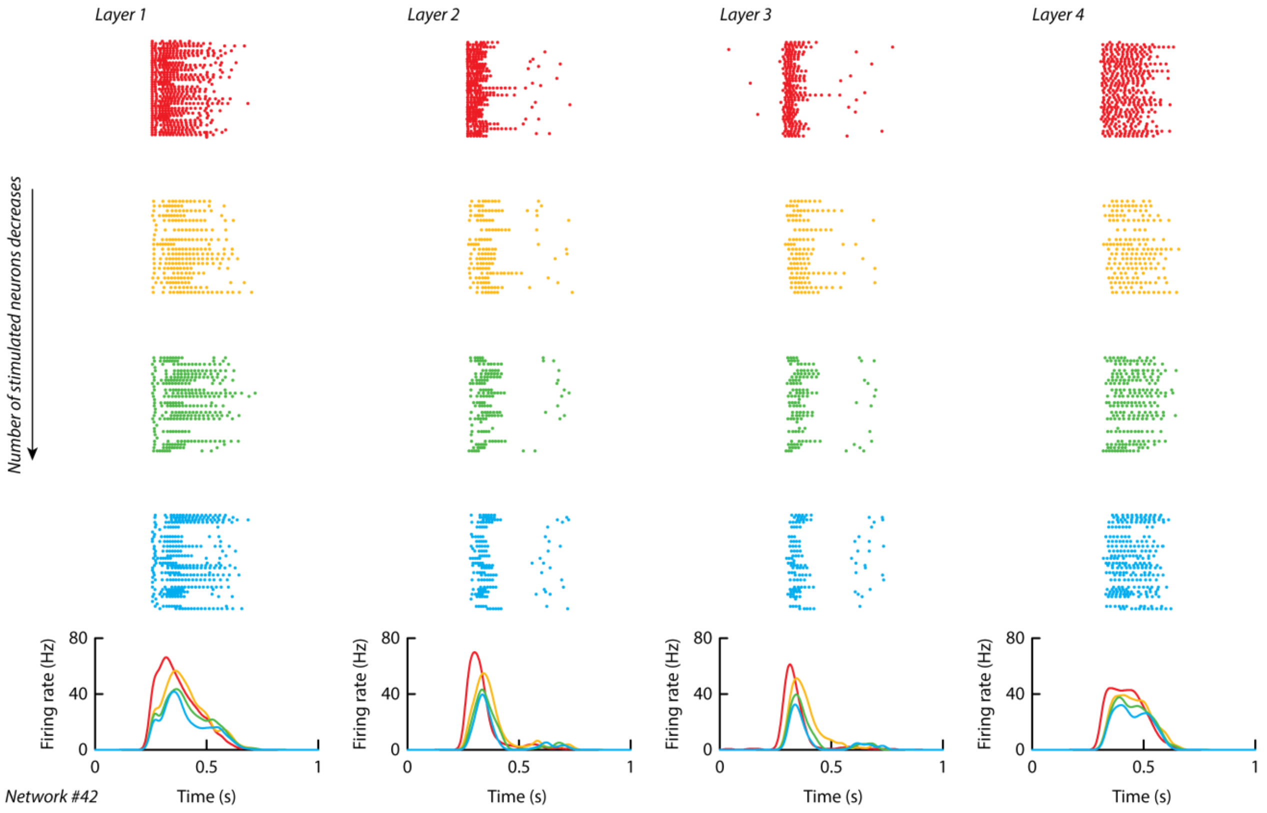

Supplementary Figure 5: Modulation of firing rate by the number of stimulated neurons.

Example of raster plots (top) and PSTH (bottom) for 4 neurons recorded in layers 1-4 in response to synchronous activation of 36 neurons selected in layer 1 (red). Activation of a subset of neurons randomly selected from this initial pool resulted in a decrease of firing rate (orange: 18 neurons; green: 12 neurons; blue: 9 neurons).

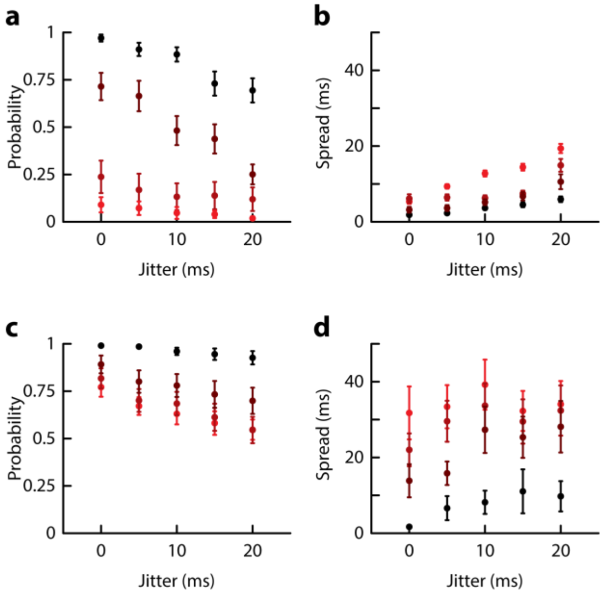

Supplementary Figure 6: Propagation of pulse packets in multilayer networks.

a, Spike probability as a function of jitter in the stimulating pulse packet for different layers in sparse networks (layer 1 to layer 4, from black to red respectively). The spike probability was defined as the probability of evoking at least 1 spike in the 0.5 ms following the stimulation. **b**, Spike delay as a function of jitter in sparse networks. **c**, Same as in **a** but in dense networks. **d**, Same as in **b** but in dense networks. Data are the same as in Figs. 2-3 ($n = 15$ sparse and $n = 16$ dense networks) and are presented as mean ± SEM.

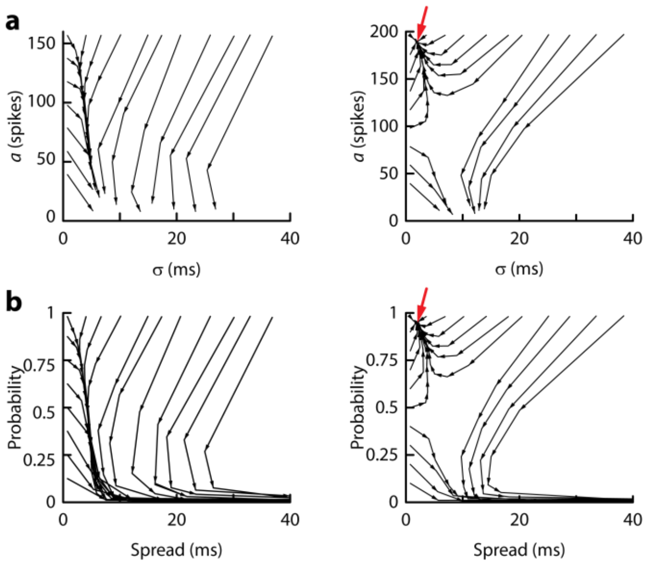

Supplementary Figure 7: Simulation of phase diagram for idealized feedforward networks.

Because we could only record from a maximum of 4 neurons, the phase diagram in Fig. 3 of the main text was constructed from the responses of a single neuron to repeated, identical stimulation rather than from the responses of a population of neurons to a single stimulus [2]. To confirm that they produce similar results, we performed the following simulations.

a, Phase diagrams constructed from the number of spikes in each layer as a function of spike spread across the population as originally defined by [2]. Two configurations were used: *left*, when the number of neurons in each layer was small, trajectories lead to extinction; *right*, when the number of neurons in each layer is larger than a critical value, trajectories lead to a fix point (red arrow) where propagation is stable.

b, Phase diagrams constructed with the same data as in **a** but from the probability of spiking in each layer as a function of spike spread across trials for a single neuron as shown in Fig. 3 of the main text.

Both representations are qualitatively similar and display the dispersion of the pulse packet when the number of neurons in each layer is small (left) and a stable attractor when the number of neurons is large (right).

Simulation parameters: Simulations were made from networks composed of 10 layers, each containing 160 (left) or 200 (right) excitatory neurons. Connection probability was 0.2 between layers and connections were exclusively feedforward. We used leaky integrate-and-fire neuron model with input resistance R_m = 160 MΩ and membrane time constants τ_m = 20 ms. The resting membrane potential was V_{rest} = -60mV and the spike threshold was V_t = -44mV. Synapses were conductance-based and at rest, EPSPs had amplitude of 2 mV.

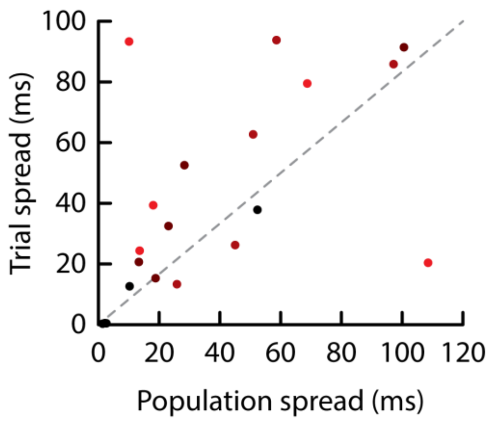

Supplementary Figure 8: Relation between trial and population measures of spike spread.

With 4 recorded neurons in a given layer (see an example in Supplementary Fig. 11), we could compute the standard deviation of the spike time from the responses of a single neuron to repeated, identical stimulation (trial spread) of from the responses of the population of neurons to a single stimulus (population spread). The color code denotes the layer from which the neurons where recorded (black to red for layer 1 to layer 4, respectively). Both estimates of the spike spread were correlated (Pearson correlation: $r = 0.57$, $P = 0.008$, $n = 20$). The grey dotted line shows the unity line.

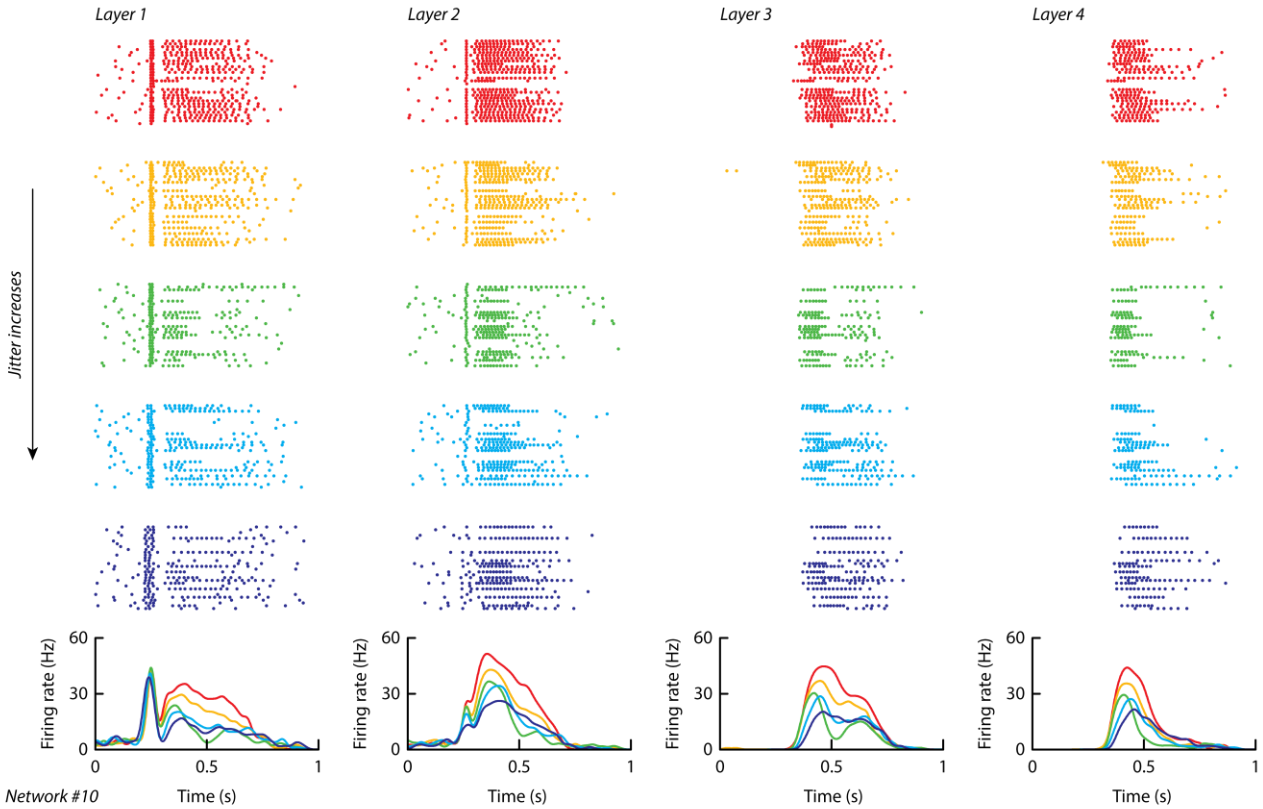

Supplementary Figure 9: Propagation of spike timing in a dense multilayer network.

Data shown in Fig. 4 of the main text expanded to show rasters obtained with all the stimulus conditions. Raster plots (top) and PSTHs (bottom, red to dark blue for 0 to 20 ms jitters) for different stimulus jitters. One neuron was recorded in each layer.

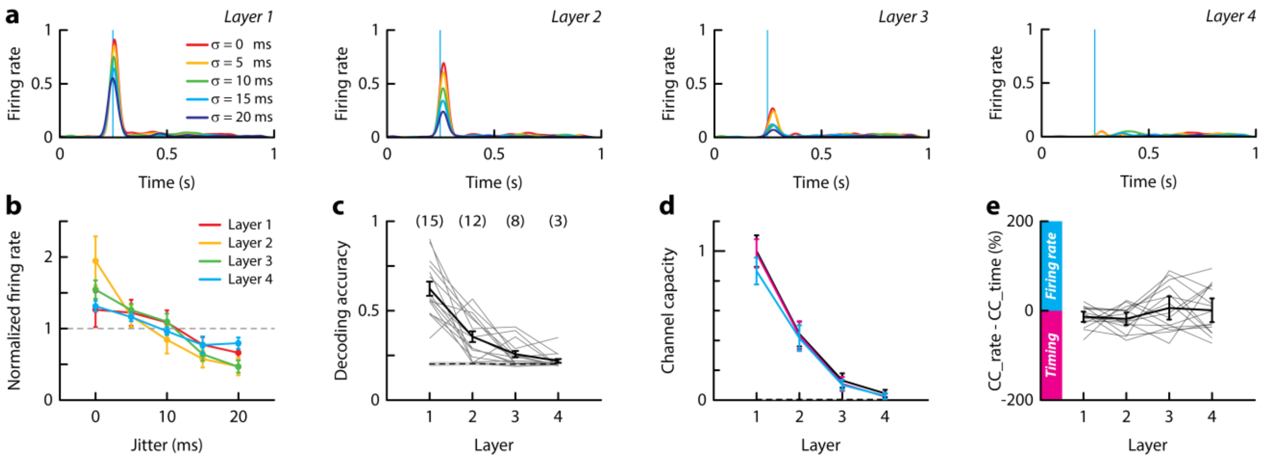

Supplementary Figure 10: Propagation of information about the stimulus jitter in sparse multilayer networks.

a, Average PSTHs for different stimulus jitters (σ) of 0 (red) to 20 ms (blue) in sparse networks. **b**, Firing rate as a function of jitter for neurons in layer 1 to 4. The firing rate measured in a 300 ms time window following the stimulus was normalized (divided) by the rate averaged over the different jitters. **c**, Decoding accuracy quantified as the probability of correctly classifying stimulus jitter in each layer. Decoding used time of the first spike and firing rate as predictors (see main text). Numbers in brackets indicate the number of individual neurons (out of 15 neurons for each layer) with significant decoding accuracy with a *p*-value of 0.01 (see Methods). **d**, Channel capacity computed using the time of the first spike and the firing rate (black) or only the firing rate (magenta) or the time of the first spike (cyan) as predictors. **e**, Difference between the information carried by the firing rate and the information carried by the time of the first spike relative to their sum. Data are from the same sparse networks as in Fig. 2 (n = 15) and are presented as mean ± SEM. In **c** and **e**, all data are shown as thin lines.

In the synfire mode, decoding accuracy and channel capacity were high in the first layers but dropped to chance level by the 4th layer because propagation mostly stopped. In contradistinction to propagation in the rate mode, information carried by the spike timing was always comparable to the one carried by the firing rate.

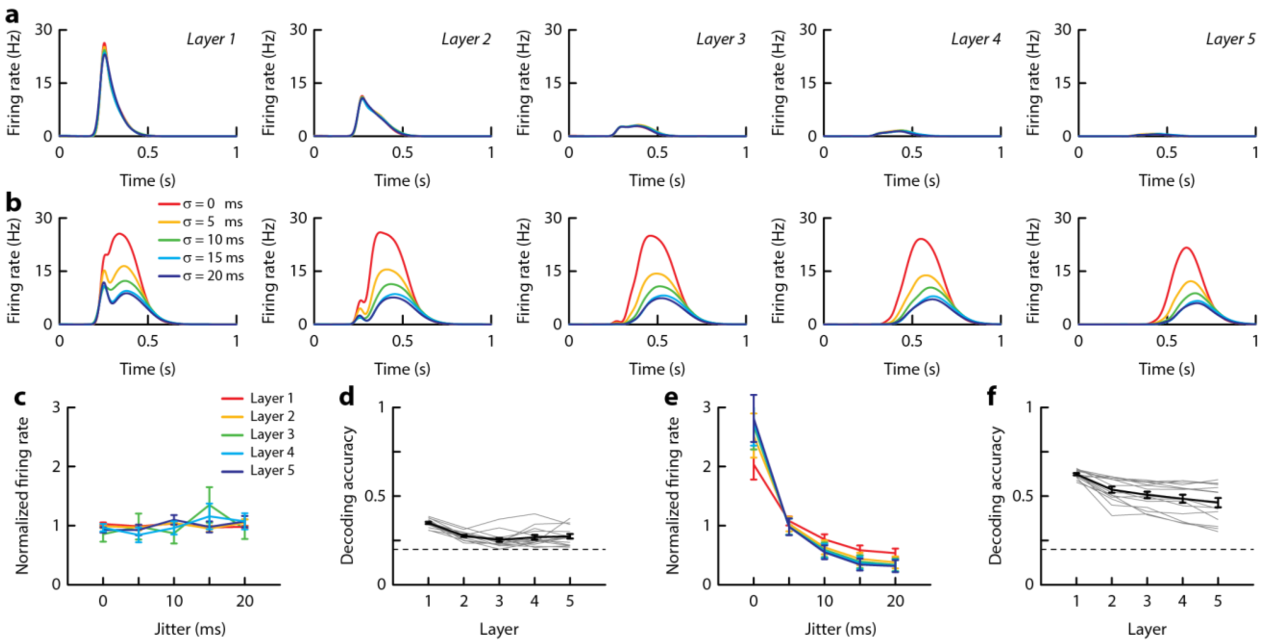

Supplementary Figure 11: Propagation of stimulus jitter in the simulated network.

a-b, Firing rate compiled from the spiking of neurons evoked with varying stimulus jitters (σ = 0, 5, 10,15, 20 ms; from red to dark blue) in sparse (**a**, 100 neurons per layer) or dense (**b**, 700 neurons per layer) networks. **c**, Firing rate as a function of jitter for neurons in layer 1 to 5 in sparse networks. The firing rate measured in a 300 ms time window following the stimulus was normalized (divided) by the rate averaged over the different jitters. **d**, Decoding accuracy quantified as the probability of correctly classifying stimulus jitter in each layer. Decoding used time of the first spike and firing rate as predictors. **e-f**, Same as **c-d** for dense networks. Data are compiled from n = 15 in sparse or dense networks and are presented as mean ± SEM. In **d**, and **e** all data are shown as thin lines.

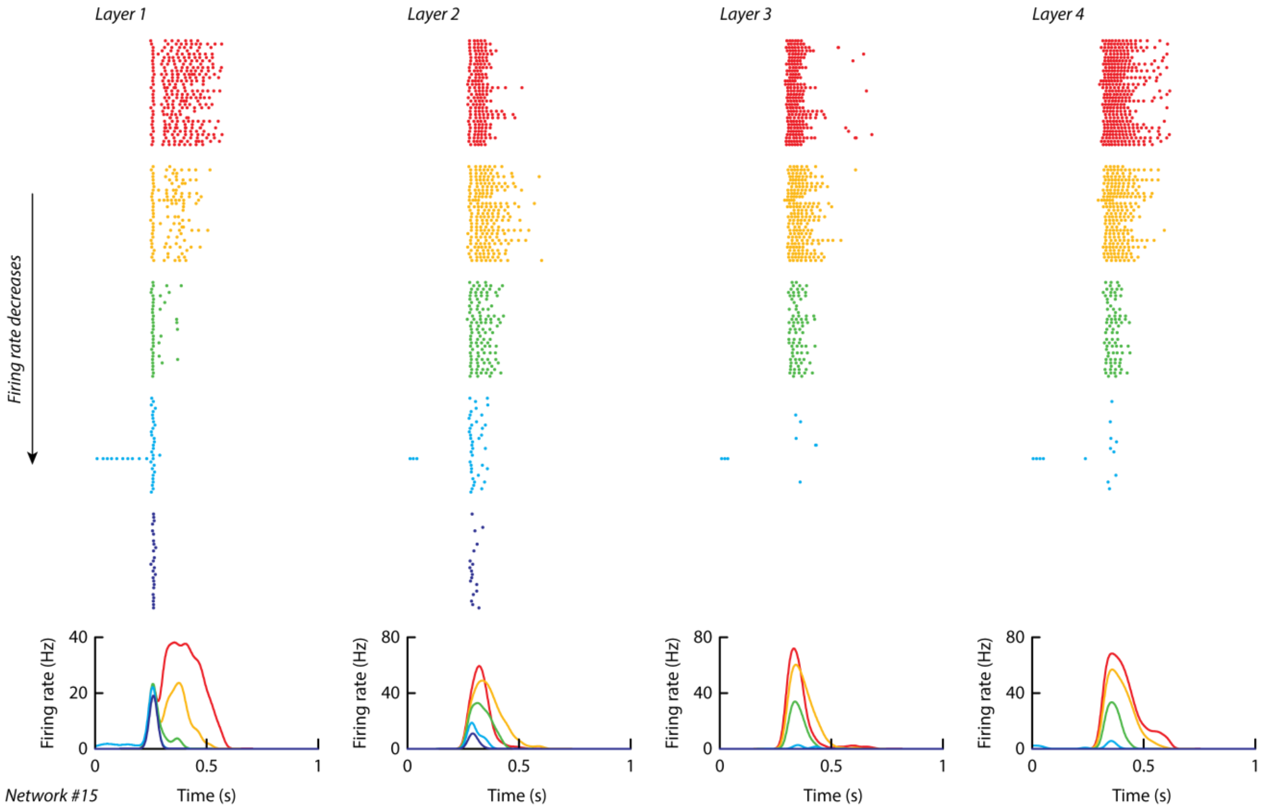

Supplementary Figure 12: Propagation of firing rate in dense multilayer networks.

Raster plots (top) and PSTH (bottom, red to dark blue for high to low firing rate in layer 1) for different firing rates in the 1st layer. One neuron was recorded in each layer. Trials were sorted according to the number of spikes in the 1st layer and binned into 5 groups (see main text and Methods).

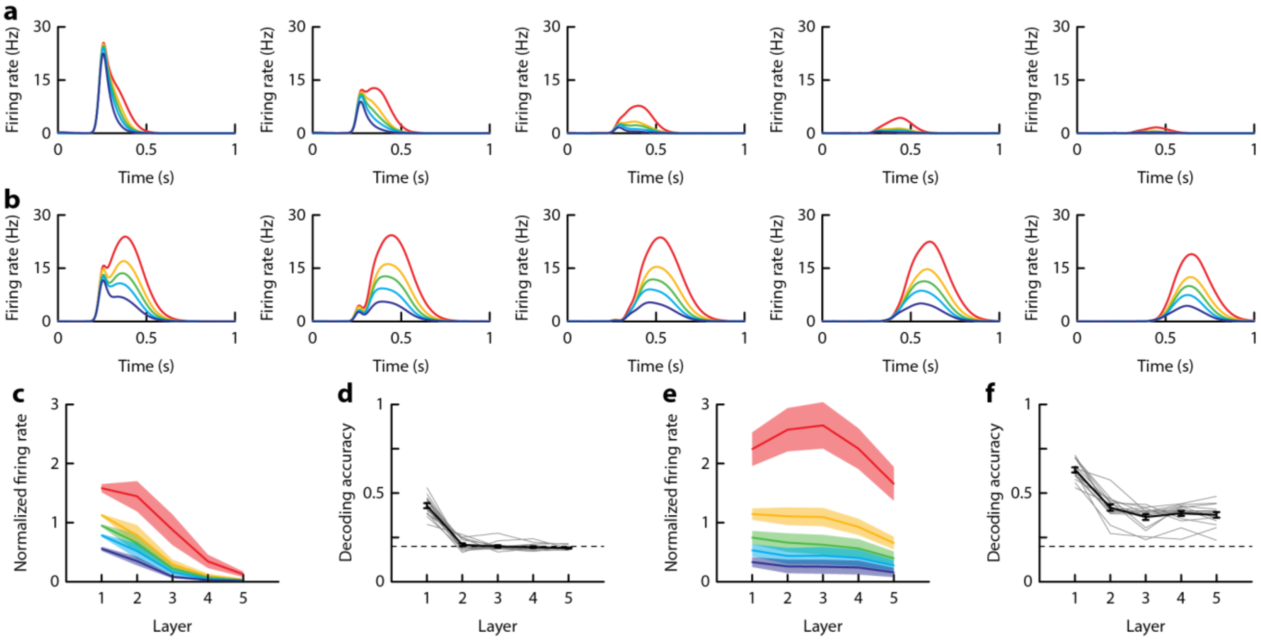

Supplementary Figure 13: Propagation of firing rate in the simulated network.

a-b, Firing compiled from trials sorted in 5 groups based on firing rates in layers 1 (see Methods) in sparse (**a**, 100 neurons per layer) or dense (**b**, 700 neurons per layer) networks. **c**, Firing rate vs layer. For each neuron of each network, the firing rate was normalized by the average rate across the different groups. **d**, Decoding accuracy quantified as the probability of correctly classifying firing rate in the first layer given the firing rate in the n*th* layer. **e-f**, Same as **c-d** for dense networks. Data are compiled from n = 15 in sparse or dense networks and are presented as mean ± SEM. In **d**, and **e** all data are shown as thin lines.

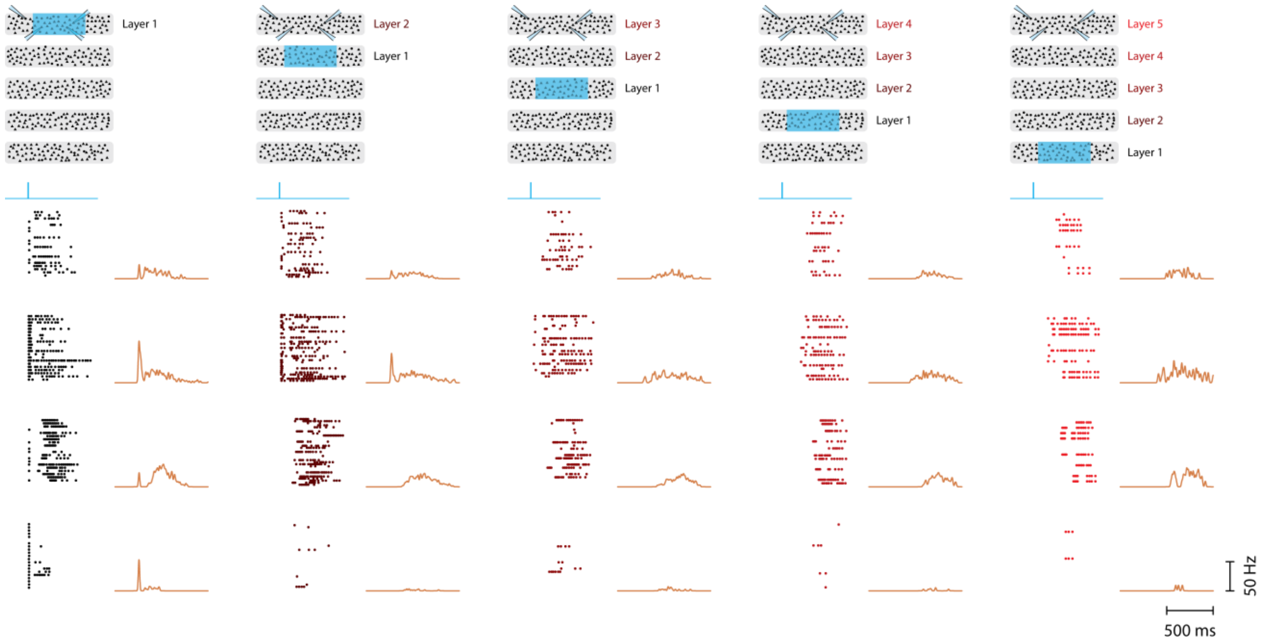

Supplementary Figure 14: Spiking activity of neurons in the same layer.

Example of raster plots (left) and PSTH (right) for 4 neurons recorded in the same layer when different layers (1 to 5) were activated by a large field synchronous stimulus.

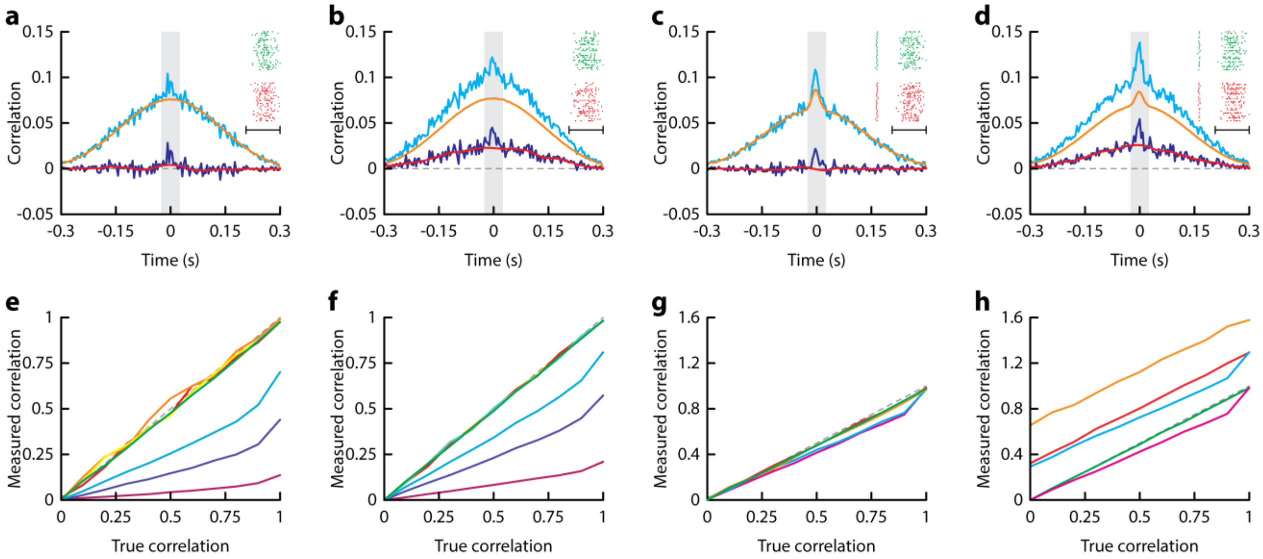

Supplementary Figure 15: Estimation of spike correlation.

a, To build an accurate measure of correlation, we simulated 2 neuronal spike trains where a fixed percentage of spikes were identical in order to vary the correlation in spikes C_{spike} between 0 and 1 (inset; the correlation C_{spike} is here 0.1; scale bar 0.5 seconds). The firing rate was not constant and had a cosine envelop with a maximum rate of 25 Hz. Synchronous and non-synchronous spikes were generated by a Poisson process. Spikes were further randomized with a stochastic jitter of standard deviation 5 ms. From this spike train, we computed the raw cross-correlogram histogram that contains both noise and signal correlations (cyan curve). To isolate the signal correlation only, we cross-correlated each trial of the first neuron with any other trial of the second neuron (orange curve). The subtraction of these two curves gives the noise correlation histogram (dark blue curve). The noise correlation was filtered using a smoothing spline fit (red curve) for which we removed the data points corresponding to an interval of length T_{int} (here $T_{int} = 50$ ms and is denoted by the grey bar) centered around $t = 0$. The correlation coefficient was calculated as the integral of the area between the noise correlation and the smoothing function in the interval [-0.3 ms, 0.3 ms]. **b**, Same as in **a** but the rate varied on the trial-by-trial basis which resulted in slow timescale noise correlation. The smoothing spline fit allowed eliminating this correlation while keeping the short timescale correlation that we wanted to evaluate. **c**, Same as in **a** but we added a single synchronous spike at the onset of each trial to simulate the experimental data and assess its effect on the correlation measure. This spike resulted in a peak of the signal correlation at short timescale. **d**, Same as in **a** but with the trial-by-trial rate fluctuation and the synchronous spike at the onset. **e**, Measured correlation using the described method with different T_{int} (5, 10, 20, 50, 100, 150, 200 ms, red to purple lines, respectively) as a function of the true correlation C_{spike} for neuronal spike trains generated as in **a**. The grey dotted line denotes the slope of unity. **f**, Same as in **e** but the correlation was simply measured as

the integral of the noise correlation (dark blue curve) in the interval [-0.3 ms, 0.3 ms]. **g**, Measured correlation using the described method with $T_{\mathrm{int}} = 50$ ms as a function of the true correlation C_{spike} for neuronal spike trains generated as in **a** (random spikes with varying C_{spike}; green), as in **b** (random spikes with varying C_{spike} and trial-by-trial rate fluctuations; red), as in **c** (random spikes with varying C_{spike} and the synchronous spike at the onset; magenta), as in **d** (random spikes with varying C_{spike}, trial-by-trial rate fluctuations and the synchronous spike at the onset; cyan), as in **b** but with a larger maximal firing rate of 50 Hz (random spikes with varying C_{spike} and trial-by-trial rate fluctuations; orange). Our measure allowed determining C_{spike} faithfully in every condition. **h**, Same as in **g** but for the simple method described in **f**. This shows that the trial-by-trial rate fluctuations lead to an overestimation of the true correlation and need to be removed.

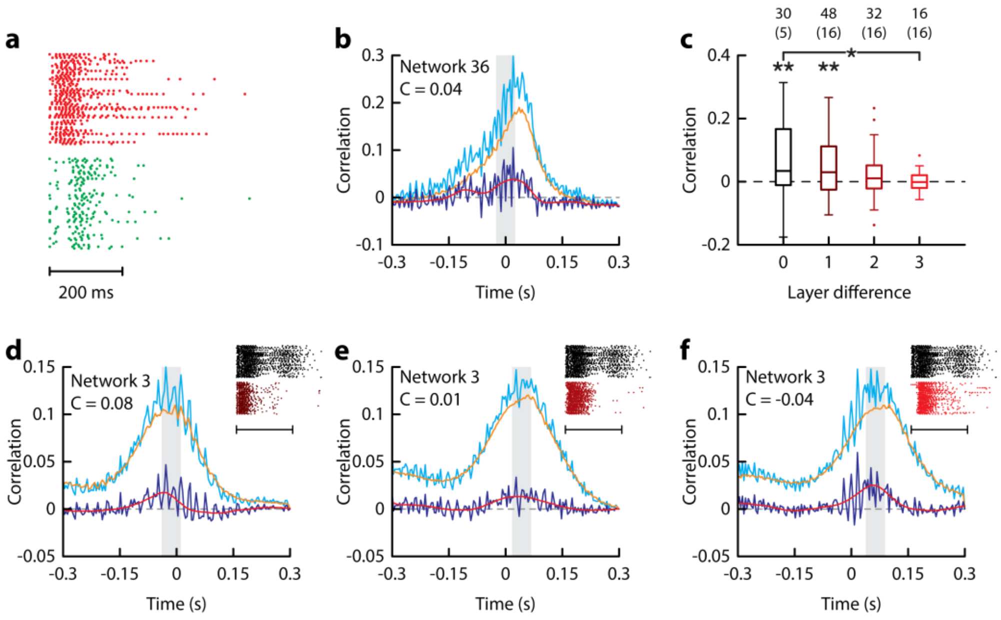

Supplementary Figure 16: Correlation within and across layers in the multilayer network.

a, Example of raster plot of spiking activity from two neurons recorded simultaneously in the same layer. **b**, Corresponding cross-correlogram with the same color code as in Supplementary Fig. 11 (raw cross-correlation, cyan; signal correlation, orange; noise correlation, dark blue; filtered noise correlation, red). **c**, Spike correlation in dense network as a function of difference in recording layers. Numbers of neuronal pairs are indicated above whisker plots (box: median and interquartile range, whiskers: full range of the distribution; outliers are plotted individually) and numbers of preparations in brackets. Box plots indicate median and interquartile range, whiskers cover the full range of the distribution and outliers are plotted individually. Statistical significance was assessed using Mann-Whitney *U*-test (the number of recorded pairs of neurons is shown above whisker plot and the number of cultures in brackets). Only significant differences are shown, * $P < 0.05$, ** $P < 0.01$. Difference between a distribution and 0: $P = 0.0034$ (C_0), $P = 0.0051$ (C_1), $P = 0.1499$ (C_2), $P = 0.7564$ (C_3); difference between 2 distributions: $P = 0.3318$ (C_0 vs C_1), $P = 0.0795$ (C_0 vs C_2), $P = 0.0217$ (C_0 vs C_3), $P = 0.3285$ (C_1 vs C_2), $P = 0.1052$ (C_1 vs C_4), $P = 0.3304$ (C_2 vs C_3). **d-f**, Examples of cross-correlograms for neurons recorded in successive layers (between L1 and L2, L2 and L3, or L3 and L4, **d**, **e**, or **f**, respectively) with the same color code as **a**.

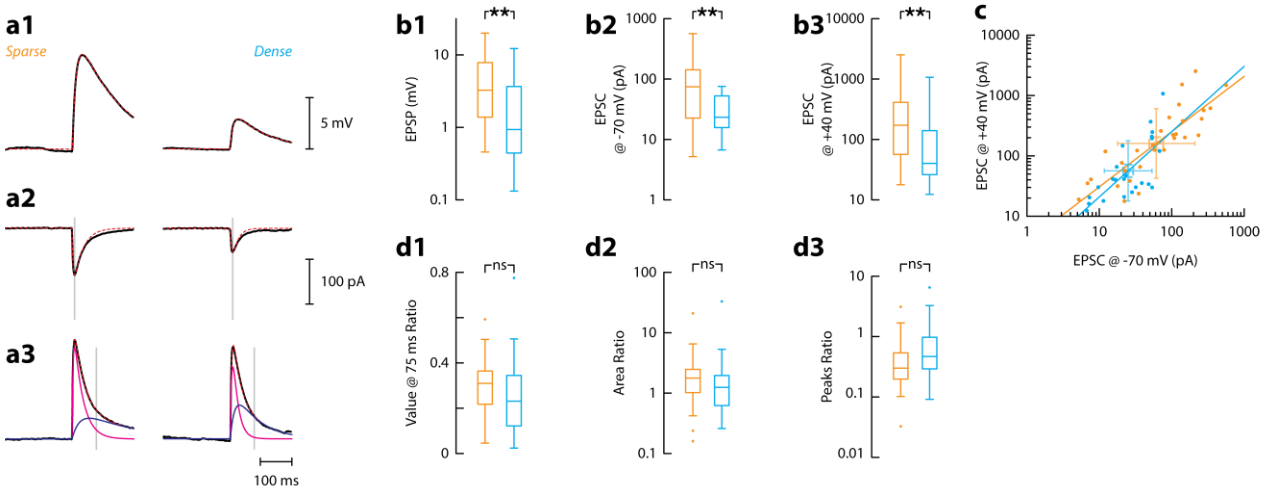

Supplementary Figure 17: NMDA/AMPA ratio in networks of different densities.

a, Representative excitatory postsynaptic potential (EPSP, **a1**) and excitatory current recorded at -70 mV ($EPSC_{-70}$, **a2**) or at +40 mV ($EPSC_{+40}$, **a3**) in sparse (left, 202 neurons·mm^{-2}) and dense (right, 920 neurons·mm^{-2}) networks. These experimental traces (black) were fitted by analytical functions (dashed red lines) to determine their peak and areas. EPSPs were fitted by an alpha function. $EPSCs_{-70}$ were fitted by a double exponential function from which we estimated a rise and a decay time. $EPSCs_{+40}$ were fitted by a sum of two double exponential functions using the two characteristic times estimated earlier as fixed parameters (AMPA current shown in magenta and NMDA current shown in dark blue). **b**, EPSP (**b1**), $EPSC_{-70}$ (**b2**), and $EPSC_{+40}$ (**b3**) sizes are represented as whisker plots (box: median and interquartile range, whiskers: full range of the distribution; outliers are plotted individually) for sparse (orange) and dense (blue) networks. These values were measured at the peak and were significantly larger in sparse than in dense networks in agreement with previous results [1, 3]. ** P <0.01; EPSP: $P = 0.0042$; $EPSC_{-70}$: $P = 0.0032$; $EPSC_{+40}$: $P = 0.0044$ ($n = 30$, 306±140 neurons·mm^{-2} for sparse networks and $n = 23$, 876±144 neurons·mm^{-2} for dense networks). **c**, The size of $EPSC_{-70}$ and $EPSC_{+40}$ were correlated (Pearson correlation coefficient in sparse networks: $r = 0.85$, $p = 3.6\times10^{-9}$, $n = 30$; and in dense networks: $r = 0.71$, $p = 1.3\times10^{-4}$, $n = 23$) and their slopes were not significantly different from unity (0.91±0.22 and 1.08±0.48 for sparse and dense networks, respectively) suggesting that NMDA and AMPA current varied proportionally. **d**, We used three measures of NMDA/AMPA ratio. *Left*: ratio between the value of $EPSC_{+40}$ at 75 ms (grey line in **a3**) and the peak value of $EPSC_{-70}$ (grey line in **a2**). *Middle*: ratio between the area of NMDA current and the area of AMPA current, both of which were estimated from the fits of $EPSC_{+40}$ (see fits in **a3**). *Right*: ratio between the peak value of NMDA current and the peak value of AMPA current, both of which were estimated from the fits of $EPSC_{+40}$ (see fits **a3**). None of these ratios

were significantly different (Mann-Whitney *U*-test statistics: Ratio 1: P = 0.14; Ratio 2: P = 0.19; Ratio 3: P = 0.051).

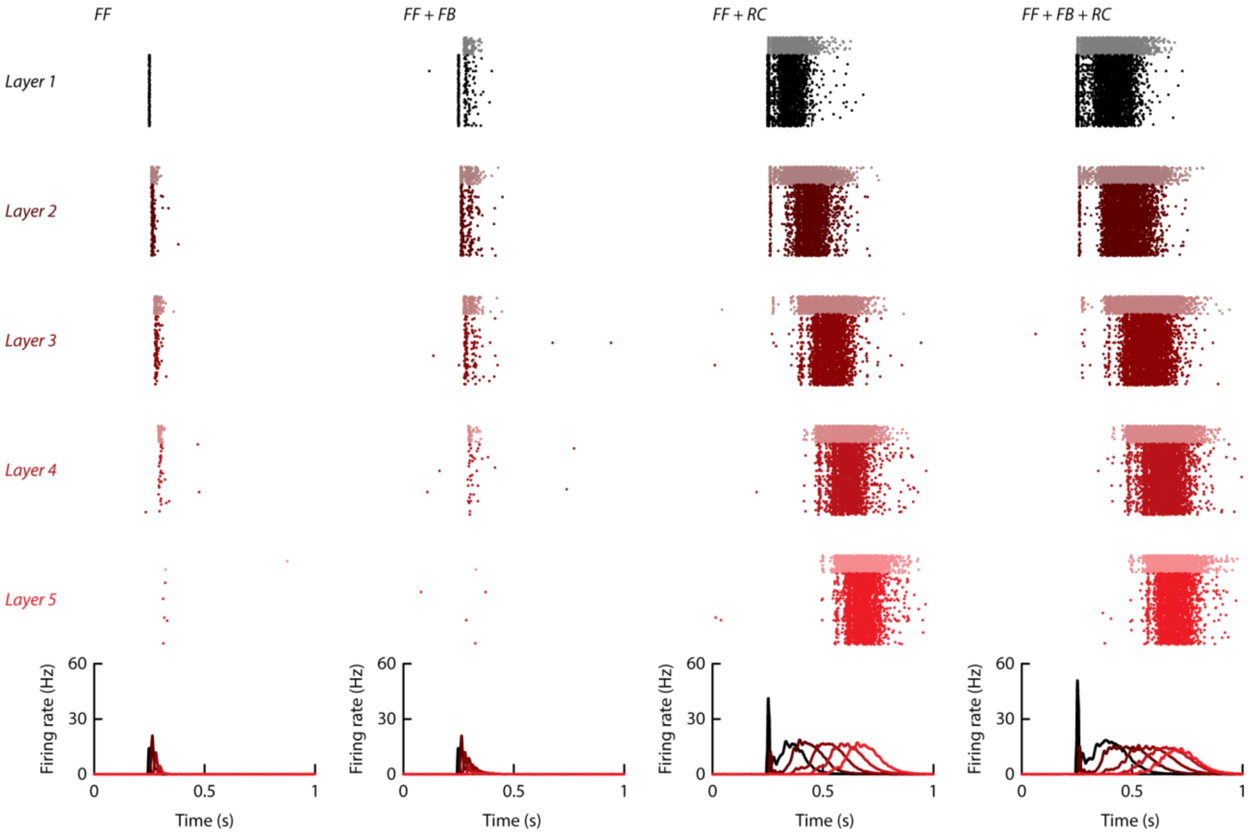

Supplementary Figure 18: Role of the different connection types in the propagation.

Simulations of a multilayer network composed of 600 neurons (480 excitatory and 120 inhibitory) in each layer. **a**, The network with only feedforward connections failed to propagate the input when inhibitory neurons were considered. **b**, Adding feedback connections across layers is not sufficient to promote propagation. **c**, In contrast, adding recurrent connections (excitatory but also inhibitory) within each layer preserved propagation via a persistent phase of activity mediated by NMDA currents. **d**, Propagation with recurrent and feedback excitatory connections is not qualitatively different from propagation with recurrent connections alone which suggest that the role of feedback excitatory connections is marginal.

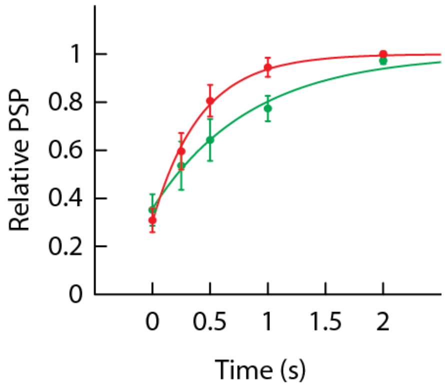

Supplementary Figure 19: Synaptic adaptation and recovery.

A train of 10 spikes at 50 Hz was elicited in the presynaptic neurons. Plotted is the ratio of the test PSP by the first PSP as a function of delay between the last spike of the train and the test spike for excitatory (green) and inhibitory (red) synapses. The data points were fitted by an exponential curve: $PSP = 1 - a \cdot \exp(-t/\tau)$, where PSP is the relative size of the post-synaptic potential, t is time, a is the amount of depression and τ is the characteristic timescale of recovery from depression. We found the following fitting parameters for excitatory (E) and inhibitory (I) connections $a_E = 0.64 \pm 0.08$, $a_I = 0.70 \pm 0.05$, $\tau_E = 846 \pm 240$ ms, $\tau_I = 415 \pm 68$ ms (coefficient with 95% confidence interval). Data are averaged from $n = 4$ excitatory connections and $n = 7$ inhibitory connections. Error bars indicate ± SEM.

Recording	Figures	Networks	Cells
Spikes	2, 3, 4, and 5	31<br>*15 sparse - 16 dense*	124<br>*60 sparse – 64 dense*
V_m	6	11<br>*6 sparse - 5 dense*	44<br>*24 sparse – 20 dense*
Spikes	7	9	36
Spikes	8	11	44
Connections	S2	13	51<br>*150 connections tested*
Spikes	S4	5	20
Spikes	S14	5	20
EPSP	S17	30<br>*15 sparse – 15 dense*	53<br>*30 sparse – 23 dense*
Total		**115**	**392**

Supplementary Table 1: Data collection

Table summarizing the number of neurons and the number of experiments (i.e. the number of networks) that were used in each figure. The first column indicates the type of recording, the second the figure's number that displayed the data and the additional columns indicate the number of networks and the number of neurons that were used for sparse and dense networks. Except for the measure of connection probability in Supplementary Fig. 2 and for the characterization of EPSP in Supplementary Fig. 17, 4 neurons were always recorded in a given network. One neuron was monitored in each layer except for Supplementary Fig. 14 where 4 neurons were recorded in the same layer.

References

1. Barral J, Reyes AD. Synaptic scaling rule preserves excitatory-inhibitory balance and salient neuronal network dynamics. *Nat Neurosci* **19**, 1690-1696 (2016).

2. Diesmann M, Gewaltig MO, Aertsen A. Stable propagation of synchronous spiking in cortical neural networks. *Nature* **402**, 529-533 (1999).

3. Ivenshitz M, Segal M. Neuronal density determines network connectivity and spontaneous activity in cultured hippocampus. *J Neurophysiol* **104**, 1052-1060 (2010).
